# From Design to Application: Advanced Cellulose Scaffolds for Engineered Tissue Regeneration

**DOI:** 10.3390/polym18050614

**Published:** 2026-02-28

**Authors:** Yao Tong, Yong Cai, Yanting Wu, Wenkun Zhuo, Jinfeng Liao

**Affiliations:** 1 State Key Laboratory of Oral Diseases & National Center for Stomatology & National Clinical Research Center for Oral Diseases, West China Hospital of Stomatology, Sichuan University, Chengdu 610041, China; 2 Department of Orthopedics, the 960th Hospital of the People’s Liberation Army Joint Logistic Support Force, Jinan 250031, China

**Keywords:** cellulose, scaffolds, tissue regeneration, biomaterials

## Abstract

The regeneration of complex tissues demands advanced scaffolds that offer biomimetic support and tissue-specific bioactive guidance. However, the materials in clinic face big challenges with immune rejection, limited donors, and unsatisfactory inductive activity. Fortunately, cellulose-based scaffolds have risen as a leading sustainable platform, considering their natural abundance, inherent biocompatibility, and highly tunable properties. This review comprehensively presented their evolution from rational design to potential clinical application. The primary cellulose sources and key detailed engineering strategies, including chemical modification, composite formulation, and bioactive functionalization, were arranged logically. The modification of cellulose can tune the physical, chemical, and biological behavior of scaffolds, along with advanced three-dimensional printing fabrication techniques. These material advances have enabled targeted functional outcomes in preclinical models, demonstrating promise for specific applications such as wound healing and bone repair. However, their broad clinical translation is contingent upon resolving persistent challenges, including controlled biodegradation and immune compatibility, which we critically assess alongside emerging frontiers such as smart responsive systems. By bridging material innovation with clinical needs, this review may provide an integrated perspective to guide future cellulose-based scaffold design for tissue regeneration.

## 1. Introduction

The reconstruction of extensive and multilayered tissue defects, resulting from trauma, oncological resection, or chronic diseases, remains a significant and prevalent clinical challenge [1]. These defects, characterized by the compounded loss of soft and hard tissues, create a complex environment that surpasses the body’s innate regenerative capacity, often resulting in permanent functional or cosmetic impairment [2]. Traditional solutions, such as autografts and allografts, are constrained by donor site morbidity, limited availability, and risks of immune rejection or disease transmission [3]. Similarly, the long-term use of certain synthetic biomedical implants has been challenged by adverse biological reactions and complications [4], highlighting a critical unmet need for superior biocompatible materials. This clinical impasse has propelled the field of tissue engineering (TE) to the forefront of regenerative medicine. TE offers a paradigm shift by aiming to create functional biological substitutes through the integrated use of cells, bioactive signals, and scaffolds [5].

Central to this strategy is the scaffold—a three-dimensional (3D), artificial extracellular matrix (ECM) designed to be far more than a passive implant. It serves as a temporary yet instructive template that actively guides and supports the entire regeneration process [6]. Therefore, the design of an ideal scaffold must meet a multifaceted set of demands. Primarily, it must provide structural and mechanical support that biomimics the native tissue ECM, which typically necessitates a highly porous and interconnected architecture to facilitate cell infiltration, vascularization, and nutrient exchange. Concurrently, the material must exhibit excellent biocompatibility paired with an appropriate, controllable degradation profile that matches the pace of new tissue formation. Beyond these fundamental roles, an advanced scaffold should ideally possess bioactive functionality to actively orchestrate critical cellular processes, including adhesion, proliferation, and differentiation [7]. However, conventional single biomaterials often fall short of integrating the comprehensive property suite required for ideal tissue regeneration, such as balanced bioactivity, controllable degradation behavior, and precise structural biomimicry of the ECM [8]. Specifically, there are concerns about immunogenicity risks and batch differences in animal-derived materials [9], as well as the possibility that certain synthetic polymers may have insufficient biological activity or their degradation products are not conducive to good tissue integration. Consequently, there remains an urgent need for a versatile, sustainable, and highly tailorable scaffold platform capable of bridging these functional gaps.

Addressing these multifaceted demands requires a material platform that is not only biocompatible and sustainable but also exhibits exceptional tunability across chemical, physical, and biological dimensions. Cellulose, the most abundant biopolymer on Earth, has emerged as a leading sustainable candidate. Derived from diverse sources such as plants, marine algae, and acetic acid bacteria, cellulose offers inherent advantages, including natural abundance, excellent biocompatibility, and biodegradability [10]. Its immense potential to evolve from a passive structural material into a dynamic platform stems from its exceptional tunability across multiple scales. At the macro-scale, traditional cellulose formats provide immediate mechanical support, while at the nano-scale, cellulose nanofibers (CNFs) and cellulose nanocrystals (CNCs) offer a unique capacity to closely mimic the fibrous nano-topography of the native ECM, directly influencing cell behavior [11,12,13]. Among these nanoforms, bacterial cellulose (BC) stands out. Biosynthesized by microorganisms such as *Komagataeibacter xylinus*, BC possesses exceptional properties (with high purity, crystallinity, and superior hydrated strength) arising from its unique synthesis pathway [14,15]. This structural hierarchy, from nano to macro, forms the foundation upon which advanced functionality can be built.

The transition of cellulose from a promising raw material to a precision tool for tissue regeneration is enabled by deliberate “design-to-application” engineering strategies. Its chemical structure, rich in hydroxyl groups, provides a handle for various modifications—such as phosphorylation or sulfonation—to alter hydrophilicity, degradation rates, or introduce sites for further bio-conjugation [9,16]. Furthermore, cellulose can be seamlessly integrated into composite systems, combining with polymers like polylactic acid or bioactive inorganic phases like hydroxyapatite, to synergistically enhance mechanical properties, bioactivity, and mimic the composite nature of tissues like bone [17,18]. Perhaps most transformative is the ability to functionalize cellulose scaffolds with bioactive molecules, peptides, or drugs, empowering them to actively promote specific regenerative events, such as M2 macrophage polarization for immunomodulation, osteogenic differentiation [19], or guided neurite outgrowth [20]. Concurrently, advanced fabrication techniques like 3D printing and electrospinning allow the translation of these engineered materials into scaffolds with precise, pre-defined architectures, from patient-specific bone grafts with controlled pore connectivity to aligned nanofiber conduits for peripheral nerve repair [21,22]. Collectively, through chemical tailoring, composite formulation, bioactive functionalization, and precision manufacturing, cellulose is transformed into a programmable platform capable of meeting specific tissue regeneration challenges.

**Figure 1:**
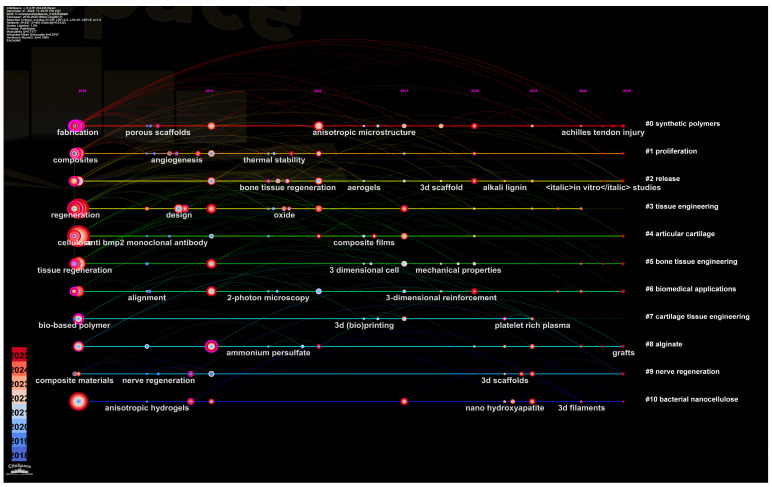
Temporal evolution of cellulose scaffold research in tissue engineering (2018–2025). The search terms were as follows: ((“cellulose” AND “scaffold*”) OR (“cellulose-based scaffold*”)) AND (“tissue regeneration” OR “tissue engineering”) AND “biomaterial*”. The language is limited to English, and the citation index is selected from the Web of Science Core Collection for the period 2018 to December 2025. A total of 336 articles were obtained. CiteSpace 6.3. R1 software was used to visualize the following data. Colored straight lines: Represent distinct research topic clusters. Curved lines: Indicate associations between two research topics. Node color: Denotes the research year. Node size: Reflects the extent of research attention on the topic. “#” followed by text: Labels the name of each research topic cluster.

**Figure 2:**
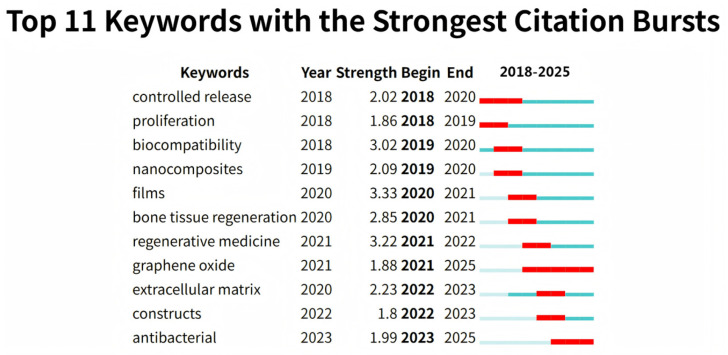
Top 11 bursting keywords in cellulose scaffold literature. The search terms were as follows: ((“cellulose” AND “scaffold*”) OR (“cellulose-based scaffold*”)) AND (“tissue regeneration” OR “tissue engineering”) AND “biomaterial*”. The language is limited to English, and the citation index is selected from the Web of Science Core Collection for the period 2018 to December 2025. A total of 336 articles were obtained. CiteSpace 6.3. R1 software was used to visualize the following data. The blue line indicates the time interval, and the red line indicates the period when the keyword burst occurs. “Burst words” refer to words that are frequently cited over a period of time.

**Figure 3:**
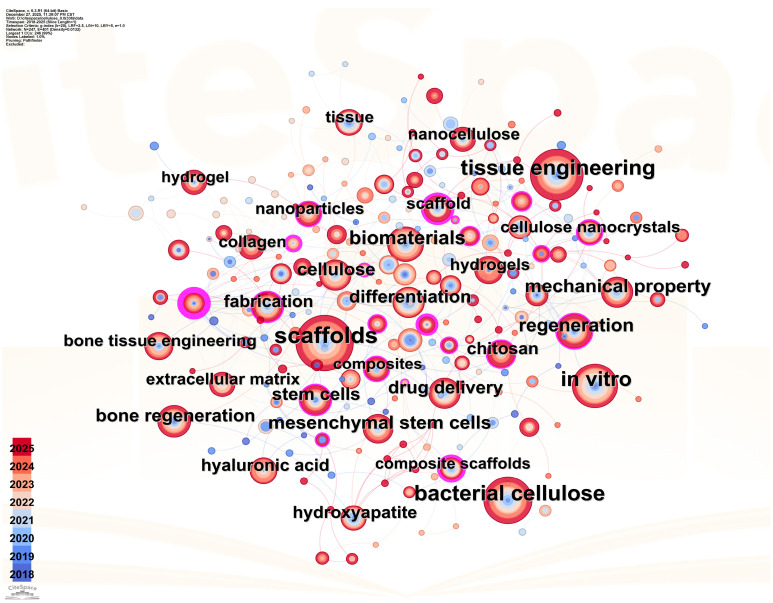
Keyword co-occurrence network in cellulose scaffold research. The search terms were as follows: ((“cellulose” AND “scaffold*”) OR (“cellulose-based scaffold*”)) AND (“tissue regeneration” OR “tissue engineering”) AND “biomaterial*”. The language is limited to English, and the citation index is selected from the Web of Science Core Collection for the period 2018 to December 2025. A total of 336 articles were obtained. CiteSpace 6.3. R1 software was used to visualize the following data. Nodes represent keywords, where node size reflects the frequency of keyword occurrence in literature; node colors indicate publication years (refer to the left-hand color legend for the year range 2018–2025); lines between nodes show the strength of co-occurrence.

The dynamic trajectory of cellulose scaffold research, quantitatively captured by literature analysis (Figure 1, Figure 2 and Figure 3), validates and contextualizes the “design-to-application” framework central to this review. Analysis reveals a clear, phased progression (Figure 1): from foundational work on porous architecture and 3D printing (2018–2020), to a maturation phase emphasizing composite scaffolds for bone regeneration (2021–2023), culminating in the current focus (2024–2025) on functional grafts and clinical translation. This evolution from structural exploration to application-specific optimization directly informs our review’s organization, which systematically traces the translation of material design into functional outcomes across tissue types.

The keyword burst analysis (Figure 2) underscores a strategic pivot in the field toward overcoming persistent clinical bottlenecks. The intense, recent bursts of “Antibacterial” (2023–2025) and “Extracellular matrix” (2022–2023) signal a critical transition: having established foundational mechanical competence, current research focuses on endowing scaffolds with sequential bioactivity to combat infection and recapitulate the native regenerative microenvironment. These emerging priorities directly motivate our in-depth discussion on bioactive functionalization strategies and their targeted deployment in soft wound healing and hard tissue repair applications.

Furthermore, the keyword co-occurrence network (Figure 3) maps the interdisciplinary logic that underpins this review. “Cellulose” serves as the central hub, with strong conceptual linkages to “3D printing”, “Drug delivery”, “Stem cells”, and “Mechanical properties”. This interconnectedness reinforces our thesis that advanced cellulose scaffolds are defined not by a single property, but by the synergistic integration of material selection, precision engineering, and advanced fabrication, a synergy methodically detailed in the following sections. Notably, while the network displays a robust research ecosystem, the comparative under-representation of high-strength burst keywords related to “biodegradation control” and “immune compatibility” prior to 2025 points precisely to the persistent translational hurdles that form a critical focus of our concluding perspective.

Thus, this bibliometric panorama not only confirms the timeliness of this review but also charts its intellectual course: tracing the evolution from engineering tunability to targeted functionality and ultimately to confronting the persistent hurdles on the path to clinical translation. More than merely mapping trends, this analysis reveals a critical tension within the field’s trajectory. While current research strongly focuses on overcoming specific clinical bottlenecks—such as equipping scaffolds with antibacterial properties and biomimetic ECM features—the keyword co-occurrence network simultaneously indicates that foundational translational challenges, including controlled biodegradation and immune compatibility, have not yet received commensurate research emphasis.

This review is structured to provide a comprehensive and balanced perspective. To achieve this integrated view, we first establish the material foundation and engineering toolkit. We begin by systematically presenting the material sources and key engineering strategies in chemical modifications, composite designs, and bioactive functionalizations. Building upon these foundational engineering strategies, the subsequent discussion demonstrates how these material advances are translated into targeted functional outcomes across key tissue regeneration domains. In the realm of hard tissue engineering, cellulose-based composites address the dual requirements of structural support and bioactive mineralization. For soft tissues, applications range from skin wound healing—capitalizing on the unique properties of BC—to nerve repair, where precisely engineered microchannels in CNF scaffolds guide axonal regrowth. Applications in specialized tissues such as cartilage and cardiovascular structures are also explored. Finally, we critically evaluate the persistent translational hurdles that currently separate these promising platforms from widespread clinical adoption, aiming to present a clear-eyed view of the path from bench to bedside.

## 2. Material Sources and Engineering Strategies

Tissue regeneration requires more than passive structural support; it necessitates a bioactive, biomimetic, and mechanically tailored microenvironment that actively guides cellular processes [23]. Within this paradigm, cellulose has emerged as a leading sustainable biomaterial platform [24]. However, its native form often lacks the dynamic properties required for specific tissue regeneration [25]. Transforming raw cellulose into advanced functional scaffolds, therefore, demands a systematic engineering approach that spans from selecting the appropriate source material to executing molecular design and macroscopic fabrication [26].

### 2.1. Cellulose Sources for Tissue Engineering: From Macro to Nano

Cellulose used in TE is derived from diverse sources, each offering distinct structural forms and properties that influence scaffold design [27]. Plant-derived cellulose, the most abundant, can be processed into traditional macroscopic forms such as woven fabrics and non-wovens for immediate mechanical support, or down into nanoscale materials like cellulose nanofibrils (CNFs) and CNCs. These nanocelluloses provide a high surface area and the ability to mimic the nanotexture of the natural ECM [28]. BC, synthesized by microbes such as *Komagataeibacter xylinus*, presents a distinct paradigm [29]. In contrast to plant-derived cellulose, BC is inherently produced as a nanofibrous network free of lignin and hemicellulose, resulting in exceptional purity and crystallinity [30,31]. This unique biosynthesis yields a seamless, mechanically robust 3D network in situ [32], which grants BC superior wet-state strength, outstanding water retention [33], and often enhanced biocompatibility compared to its plant-based counterparts, making it an exemplary “temporary matrix” for cell growth [34]. Other sources encompass algal cellulose and regenerated cellulose derivatives, such as carboxymethyl cellulose and cellulose acetate, which provide tunable solubility and processability [35,36].

The selection of material sources dictates their initial scale—whether macroscopic or nanoscale—as well as their inherent properties. Importantly, these scales are not mutually exclusive; nanocelluloses such as CNFs, CNCs, and BC are often incorporated as reinforcing or functional components within macroscopic scaffolds. For example, one study illustrates the spray-assisted co-deposition of BC nanofibers—cultured as a bulk hydrogel—with other biomass-derived nanofibers like CNFs. Through hydrogen bonding and electrostatic interactions, the integrated nanofibers enhance the mechanical integrity of the macroscopic network while concurrently introducing functional properties such as antibacterial activity and dye adsorption. This case exemplifies how nanocelluloses can be seamlessly embedded into larger-scale systems to achieve combined structural and functional gains. Such a foundational grasp of source selection and multi-scale integration underpins subsequent advances in molecular engineering and advanced manufacturing for the development of functional scaffolds [37].

**Figure 4:**
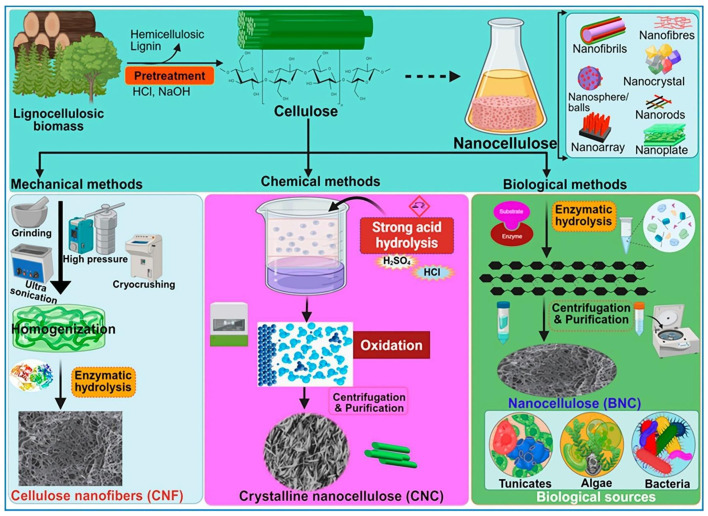
A schematic overview of the methods used for the synthesis of nanocellulose. The diagram illustrates key stages in the production process, highlighting techniques and procedures involved in deriving nanocellulose from raw materials [2]. Reprinted under the Creative Commons (CC) License (CC BY 4.0).

The selection of cellulose source dictates not only its inherent structural and mechanical properties, but also influences the subsequent processing routes for deriving nanocelluloses. These nanocelluloses, including CNFs, CNCs, bacterial nanocellulose (BNC), nanowhiskers, and nanorods, are typically obtained through various top-down or bottom-up approaches that selectively break down amorphous regions while preserving crystalline domains (Figure 4). As illustrated, methods such as acid hydrolysis, enzymatic treatment, mechanical disintegration (e.g., grinding, high-pressure homogenization), and bacterial biosynthesis are employed to yield nanocellulose with distinct morphologies and crystallinity indices, each suitable for specific biomedical applications [2].

### 2.2. Key Design Strategies for Functional Enhancement

The transformation of raw cellulose into a functional tissue engineering scaffold begins at the molecular and composite levels. Although native cellulose offers excellent biocompatibility, its performance in complex regenerative scenarios is often limited by inadequate wet-state strength, unsuitable degradation profiles, lack of cell-instructive signals, and poor conductivity for electroactive tissues. To overcome these limitations and evolve cellulose from a passive biopolymer into an active regenerative platform, three core design strategies are employed: chemical modification, composite formulation, and bioactive functionalization. These strategies are frequently combined to achieve multifunctional scaffolds that meet specific physiological demands [38].

Chemical modification fundamentally alters cellulose’s physicochemical properties by introducing new functional groups onto its backbone, primarily via reactions at the hydroxyl sites. A key example is oxidation, such as the TEMPO-mediated process, which converts primary hydroxyls to carboxylate groups [39,40]. This enhances hydrophilicity, colloidal stability, and reactivity, providing sites for ionic crosslinking or further conjugation [41,42]. For instance, carboxylated CNCs serve as nano-reinforcing phases and crosslinking points in alginate-based bioinks, where the introduced carboxyl groups strengthen hydrogen bonding and electrostatic interactions and enable more effective ionic crosslinking with calcium ions, directly enhancing mechanical strength [43]. Esterification, such as in cellulose acetate production, reduces hydroxyl density, increasing hydrophobicity and improving processability for techniques like electrospinning, while slowing enzymatic degradation [44,45]. Grafting polymerization can also introduce stimuli-responsive properties, such as thermosensitive swelling behavior [46]. These chemical modifications optimize scaffold performance on multiple fronts. They achieve this by modulating mechanical properties through crystallinity and bonding adjustments, by tuning surface wettability to control protein adsorption and cell adhesion [9,47], and by tailoring degradation rates via altered susceptibility to hydrolysis or enzymatic cleavage [48].

However, the biocompatibility of any chemical modification must be rigorously evaluated, as introduced functional groups or residual reagents can elicit unintended cellular responses [1,49]. A notable example is oxidized regenerated cellulose (ORC, e.g., Tabotamp^®^/Surgicel^®^), which contains 18–21% carboxyl groups. In a study by Leisz et al., ORC was found to induce a sharp local pH drop to approximately 1–2 upon hydration. This highly acidic microenvironment caused significant cytotoxicity, leading to rapid detachment and death of neuronal cells, astrocytes, and Schwann cells in vitro. The authors concluded that ORC may be unsuitable as a hemostatic agent near sensitive brain regions due to these pronounced pH-driven adverse effects. This case underscores that even commonly employed chemical modifications must be carefully assessed within specific biological contexts, as they can provoke detrimental cellular responses [50]. Extending from this principle, the biological implications of other common chemical modification strategies must be similarly scrutinized. For example, while phosphorylation and sulfonation are effective in mimicking ECM components to promote mineralization or M2 macrophage polarization, the introduction of such strongly anionic groups can significantly alter surface charge density. This alteration may lead to non-specific interactions with serum proteins or cell membranes, potentially resulting in unpredictable protein adsorption profiles or unintended activation of immune pathways, which requires careful evaluation within the target physiological context [16]. Similarly, TEMPO-mediated oxidation, a widely used method to introduce carboxyl groups for improved hydrophilicity and further conjugation, must be precisely controlled. Excessive oxidation can compromise the crystalline structure and mechanical integrity of CNFs [51]. Therefore, the selection of any chemical modification strategy should be guided not only by the desired functional outcome but also by a proactive and thorough evaluation of its biological implications. This necessitates comprehensive in vitro and in vivo biocompatibility testing tailored to the specific application and target tissue.

**Figure 5:**
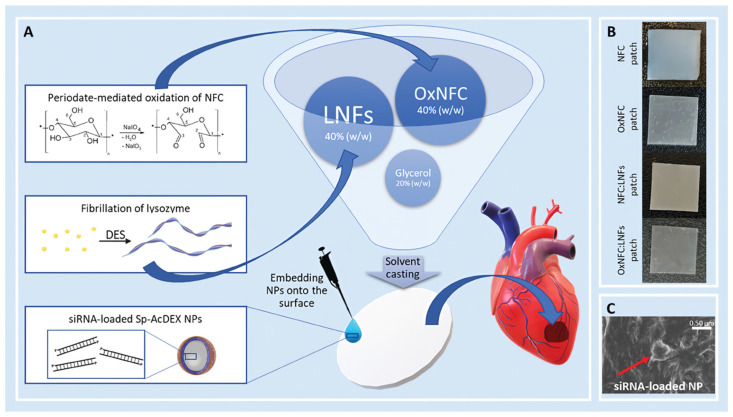
(**A**) Illustrative representation of the patch preparation intended for myocardial regeneration applications, and posterior embedding of the siRNA-loaded Sp-AcDEX NPs. (**B**) Photos of the patches (1 cm^2^ samples), from top to bottom: NFC, OxNFC, NFC: LNFs, and OxNFC: LNFs patch. (**C**) Sp-AcDEX NP embedded on the surface of a patch (scale bar is 0.5 µm) [62]. Reprinted under the Creative Commons (CC) License (CC BY 4.0).

While chemical modification fine-tunes cellulose itself, composite formulation leverages synergy between cellulose and other materials to create hybrid systems where the whole exceeds the sum of its parts [52]. The goal is to integrate complementary properties for a balanced set of characteristics. A major direction is blending cellulose with other natural polymers. Combining cellulose with gelatin, chitosan, or hyaluronic acid incorporates inherent bioactivity (e.g., RGD sequences) and enables co-crosslinking strategies that refine mechanical and degradation behavior [53,54]. For example, a hydrogel of carboxymethyl cellulose, carboxymethyl chitosan, and gelatin, crosslinked with EDAC, demonstrated enhanced stability and wound healing efficacy [55]. Blends of silk fibroin and hydroxypropyl cellulose leverage hydrogen bonding to induce a conformational shift, forming a dual-network hydrogel with superior strength and elasticity [56]. To bolster mechanical performance for load-bearing applications, cellulose is often combined with synthetic polymers like poly(lactic acid) or poly(ε-caprolactone). PLA/CNF composites prepared via salt leaching exhibit improved compressive strength and modulus while mitigating PLA’s inherent hydrophobicity, thereby enhancing cell affinity [12,57]. Incorporating CNCs into polyhydroxybutyrate-chitosan matrices increased tensile strength by 33.43% and modulus by 16.49%, alongside improved hydrophilicity [58]. For bone tissue engineering, integration with inorganic phases is critical. Compositing with hydroxyapatite (HA), bioactive glass, or amorphous calcium phosphate mimics the mineral component of bone, providing osteoconductivity, enhancing compression resistance, and promoting osteogenic differentiation [59,60]. Nanocellulose’s hydroxyl-rich surface offers favorable nucleation sites for biomimetic mineralization, as seen in the enzymatic deposition of HA on TEMPO-oxidized BC nanofibers [61]. Beyond these structural and osteogenic applications, functional composites are also designed for specialized physiological environments. A notable example is in myocardial regeneration, which demands a balance of mechanical support, bioactivity, and controlled resorption. Here, cellulose is often functionalized and combined with other bioactive components. For instance, the work of Carvalho et al. developed a self-crosslinkable patch by blending periodate-oxidized nanofibrillated cellulose (OxNFC) with lysozyme amyloid nanofibrils (LNFs). This synergistic combination leverages periodate oxidation to enhance bioresorbability and enable crosslinking, while the protein nanofibrils contribute antioxidant activity and mechanical reinforcement. The resulting OxNFC-LNFs patch exhibits enhanced wet-state mechanical properties, antioxidant capacity, and controlled degradability (Figure 5), offering a comprehensive functional profile suitable for cardiac repair [62].

**Figure 6:**
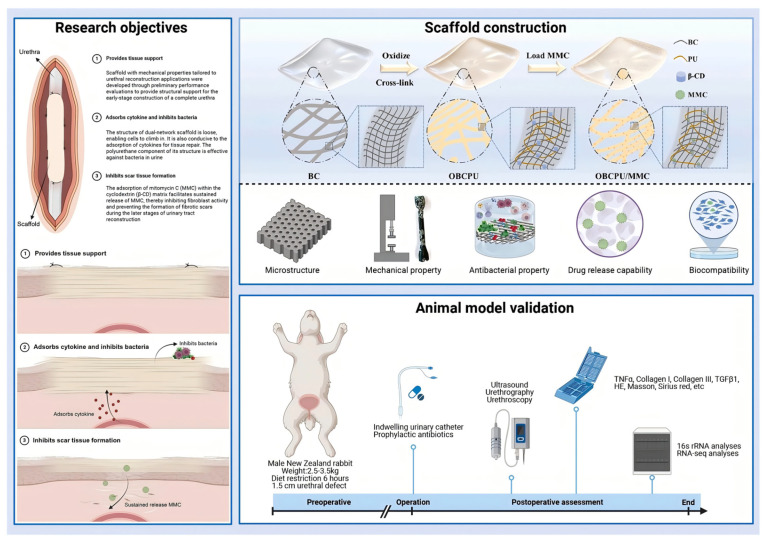
Schematic illustration of the fabrication process for a multifunctional bacterial cellulose (BC)-based scaffold for urethral regeneration [70]. The process involves in situ biosynthesis of BC, followed by chemical modification with cationic polyurethane (PU) micelles and cyclodextrin (CD) to confer antibacterial properties and enable sustained drug release. Reprinted under the Creative Commons (CC) License (CC BY 4.0).

Building upon the chemical handles and composite platforms established through the aforementioned strategies, bioactive functionalization represents the ultimate level of sophistication, transitioning the scaffold from a passive substrate to an active director of cellular fate and tissue morphogenesis [63]. This involves incorporating biological signals that specifically interact with cell receptors to elicit desired responses, such as enhanced adhesion, proliferation, differentiation, or immunomodulation [64,65]. Strategies range from physical adsorption to more stable chemical conjugation. Covalent immobilization via chemistries like carbodiimide coupling or click chemistry ensures bioactive motifs remain presented over extended periods [66]. Key agents include cell-adhesive peptides like RGD, grafted onto cellulose to promote integrin-mediated attachment [67], and growth factors like vascular endothelial growth factor (VEGF) for angiogenesis or *BMP-2* for osteogenesis [68], and therapeutic molecules like anti-inflammatory drugs or antibiotics to modulate the implant microenvironment and combat infection [69]. A sophisticated example is a BC-based scaffold modified with cationic polyurethane micelles and cyclodextrin (Figure 6), which provided antibacterial properties, sustained release of mitomycin C, and cytokine recruitment to promote repair [70]. Similarly, scaffolds have been functionalized with carbon dots for cell tracking [71] or with sulfonate groups to promote pro-regenerative M2 macrophage polarization [16]. The frontier lies in achieving spatiotemporal control over agent release, potentially through stimuli-responsive linkages that degrade in response to local pH, enzyme activity, or reactive oxygen species levels, thereby matching the dynamic phases of healing [72]. In the context of the cellulose nanofiber-based magnetic 3D nanonetwork wound dressing (CNF-MNWD) presented in a study, this vision is partly realized through a multi-stimuli-responsive platform. The dressing integrates pH-sensitive CNF-PEI, NIR-responsive gallic acid grafts, and temperature-responsive Pluronic^®^F-127, allowing it to adapt to wound microenvironmental changes [73].

The molecular and composite-level strategies described above—chemical modification, composite formulation, and bioactive functionalization—equip cellulose with tailored properties for specific regenerative tasks. However, these engineered materials realize their full potential only when shaped into three-dimensional constructs that recapitulate the architectural complexity of native tissues [74,75]. The translation of these functionalized materials into clinically relevant scaffolds is achieved through advanced fabrication techniques, which themselves often rely on the specific chemical or physical properties imparted during the design phase. The following section examines these pivotal manufacturing technologies for structural control and the essential post-fabrication processing that stabilizes the final scaffold architecture.

### 2.3. Fabrication Techniques for Structural Control

The translation of engineered cellulose materials into functional scaffolds necessitates manufacturing techniques that provide precise control over 3D architecture, which is a critical determinant of biological performance. A suite of advanced fabrication methods enables this structural control, broadly categorized into additive manufacturing, electrospinning, physical forming, and unique biofabrication strategies for BC. Each technique offers distinct capabilities for tailoring pore geometry, fiber alignment, porosity, and overall scaffold morphology to meet specific tissue regeneration requirements.

A primary technique is additive manufacturing, most notably 3D printing, which enables the digital, layer-by-layer construction of complex, patient-specific geometries with predefined internal architectures. Extrusion-based direct ink writing is prevalent for hydrogel-type biomaterials, including those based on cellulose [76]. Success depends on designing a printable bioink with specific rheological properties: it must exhibit shear-thinning behavior to flow through the nozzle and then rapidly recover viscosity after deposition to maintain shape fidelity [10,77]. Nanocelluloses, especially CNFs and nanocrystals, are exceptional rheological modifiers [78]. Their ability to form hydrogen-bonded networks imparts pronounced shear-thinning and viscoelastic behavior, making them ideal for 3D printable bioinks [79]. For instance, Read et al. incorporated CNCs into alginate hydrogels, which significantly enhanced their shear-thinning behavior and mechanical stability, enabling high-fidelity extrusion bioprinting. This study demonstrates that low CNC concentrations improve printing resolution and shape retention without compromising chondrocyte viability, offering promising rheological and biocompatible properties for engineering soft tissue constructs, particularly in cartilage tissue applications [80]. Printing parameters such as extrusion pressure, speed, nozzle diameter, and infill pattern directly dictate strand diameter, pore size, porosity, and layer adhesion [81,82]. However, the as-printed “green body” is often mechanically frail and may be water-soluble. Post-fabrication stabilization is therefore critical, typically involving a crosslinking step to solidify and strengthen the architecture. Chemical crosslinkers like glutaraldehyde create covalent networks, enhancing mechanical integrity and slowing degradation [17,83]. Ionic crosslinking, such as immersing carboxylated cellulose microfibril (CMF) films into FeCl_3_ solution, rapidly forms Fe^3+^-carboxyl coordination. This simple process significantly enhances material hydrophobicity and wet mechanical strength by creating a denser network, demonstrating an efficient, eco-friendly modification strategy for cellulose-based materials without complex chemical modification [18]. Photo-crosslinking, triggered by UV light in methacrylated polymers, allows precise spatial curing [84]. These treatments transform the printed shape into a stable, hydrated 3D scaffold capable of withstanding physiological stresses.

In contrast to the layer-wise building of 3D printing, electrospinning produces scaffolds that closely mimic the nanofibrous topology of the native ECM [85,86], yielding non-woven meshes of continuous fibers with diameters from microns to nanometers [87]. This high surface area-to-volume ratio is conducive to cell attachment and mass transfer [88]. The process involves applying a high voltage to a polymer solution, forming a charged jet that elongates and solidifies. Fiber morphology is influenced by solution properties, process settings, and environmental conditions [89]. Direct electrospinning of pure cellulose is challenging; a common strategy is to electrospin cellulose derivatives like cellulose acetate and subsequently regenerate cellulose via alkaline hydrolysis, restoring hydroxyl groups and biocompatibility [45]. Alternatively, cellulose can be blended with carrier polymers like poly(vinyl alcohol) [90,91]. Aligned fibers, crucial for tissues like nerves and tendons, can be produced using a rotating mandrel collector [7]. Post-fabrication stabilization is again essential. For derivative-based fibers, the regeneration step itself is a key chemical treatment [92]. For blend systems, crosslinking is often needed to prevent dissolution and enhance wet-state stability, achievable through chemical crosslinkers or physical methods [93].

For creating highly porous, sponge-like architectures, physical forming techniques such as freeze-drying (lyophilization) and phase separation are widely employed. Freeze-drying involves freezing an aqueous mixture and sublimating the ice crystals under vacuum, leaving a porous network [94]. Pore architecture can be controlled by adjusting freezing rate, temperature gradient, and solid content [95]. Anisotropic freezing can produce scaffolds with aligned microtubular channels, shown to direct neural regeneration by guiding axonal growth [96]. Phase separation, including solvent casting combined with particulate leaching, also creates interconnected pores by exploiting thermodynamic instability [97]. Scaffolds from these methods typically possess high porosity (>80%) and good interconnectivity, favorable for cell migration and vascularization, but often lack load-bearing strength and may collapse in aqueous environments [98]. Post-fabrication crosslinking, such as immersion in CaCl_2_ or glutaraldehyde solutions, consolidates the porous network, enhancing compressive strength, reducing swelling, and tuning degradation [99].

A distinct approach is offered by BC, which leverages unique biomolding capabilities. BC is unique due to its in situ biosynthesis capability [100]. It is secreted as a pure, nanofibrillar network by bacteria, allowing direct biomolding into predefined shapes like sheets, tubes, or auricles through static culture, while maintaining a seamless 3D nanofiber matrix. This bypasses traditional manufacturing steps and yields materials with exceptional wet strength and shape fidelity. Agitated cultures produce fibrous suspensions or pellets, offering different morphological starting points [101]. BC is also integrated with other technologies in hybrid approaches: its nanofibers can reinforce 3D-printable bioinks [102]; pre-formed membranes can serve as substrates for electrospinning or multilayer scaffolds [103,104,105]; and BC hydrogels can be cut, stacked, or combined with other materials to build complex assemblies [106,107]. Post-biosynthesis processing involves purification to remove bacterial cells and medium components, which is crucial for biocompatibility. While native BC is stable in water, integration with other polymers often requires interfacial bonding strategies, such as Schiff base reactions between oxidized BC aldehydes and amine-containing polymers, to create stable, dual-network composites [108,109].

The choice of fabrication technique depends on the target tissue’s structural and functional requirements. Three-dimensional printing excels at creating customized, complex scaffolds with designed macroporosity, ideal for bone defects or organ patches [110,111]. Electrospinning is unmatched in generating biomimetic nanofibrous environments to guide cell alignment, making it a top choice for skin, nerve, and ligament applications [112,113]. Freeze-drying produces highly porous sponges suitable for soft tissue fillers or cell seeding platforms [114,115]. The inherent biomolding of BC is uniquely suited for seamless, nanofibrous structures like vascular grafts, urethral repairs, or wound dressings [116]. These techniques are often combined in multi-process approaches to achieve hierarchical structures. As the field advances, techniques evolve; the progression from 3D to 4D printing introduces time as a dimension, enabling cellulose-based scaffolds that change shape or functionality in response to physiological stimuli, opening new frontiers for dynamic tissue regeneration [117]. Regardless of the method, robust post-fabrication stabilization—through chemical [118], ionic [119], or physical crosslinking [120]—remains an indispensable final step to transition engineered structures from lab prototypes to stable, implant-ready TE constructs.

The transformative journey of cellulose, from a versatile biopolymer to a precision-engineered tissue scaffold, hinges on the synergistic integration of material design and advanced manufacturing. As outlined, strategies for functional enhancement provide the molecular toolkit to tailor mechanical, interfacial, and biological properties. These engineered materials are then shaped by fabrication techniques for structural control, which define the critical 3D architecture, with post-fabrication stabilization locking in both form and function for physiological environments. This integrated design-to-manufacturing paradigm enables scaffolds with highly specific performance profiles. The diverse outcomes are exemplified in Table 1, which summarizes representative cellulose-based scaffolds, correlating their source material, functionalization strategy, fabrication method, key properties, and target tissue. This overview underscores the direct linkage between engineering choices and application-specific outcomes, setting the stage for a detailed exploration of their performance in regenerating specific tissues in the following chapter.

## 3. Applications in Tissue Regeneration

The true measure of any engineered biomaterial lies in its ability to address specific, unmet clinical needs. Cellulose-based scaffolds, empowered by the design strategies and fabrication techniques detailed in the preceding section, have demonstrated significant potential across a broad spectrum of tissue regeneration applications [121]. However, translating these versatile platforms into functional outcomes requires matching their engineered properties to the distinct physiological and mechanical demands of the target tissue. As summarized in Table 2, a comparative analysis of key performance metrics reveals this critical alignment: scaffolds for bone regeneration typically exhibit high compressive strength (on the order of MPa), whereas those for nerve or skin repair prioritize softer moduli (Kpa range) and high porosity. It is noteworthy that critical clinical parameters, such as in vivo degradation rates, are often inconsistently reported, a gap that highlights the need for standardized characterization in future studies. This section systematically summarizes the translation of these versatile scaffolds from the laboratory bench to functional outcomes, highlighting how purpose-driven engineering enables them to meet the distinct physiological and mechanical demands of diverse tissues. The discussion is organized by target tissue: for hard tissues like bone, the focus is on robust mechanical support and osteogenic activity [121]; for soft tissues such as skin and nerves, priorities are hydration management, barrier function, and topographical guidance [122]; and for specialized tissues including cartilage, ligament, and cardiovascular systems, the main challenge shifts to meeting unique structural and functional requirements [123,124].

### 3.1. Hard Tissues: Bone and Osteochondral Repair

The regeneration of critical-sized bone defects resulting from trauma, tumor resection, or congenital disorders remains a formidable clinical challenge [128,129]. Native bone is a complex composite material, requiring a scaffold to fulfill a multifaceted role: it must provide immediate mechanical support under load, facilitate vascular invasion, and actively promote the migration, proliferation, and osteogenic differentiation of progenitor cells [130]. The challenge is particularly demanding in osteochondral defects, which involve damage across the critical cartilage–bone interface [131]. Regenerating this complex junction requires a scaffold to simultaneously address two distinct biological and mechanical environments: the overlying articular cartilage and the underlying subchondral bone [132]. This necessitates sophisticated biphasic or gradient designs that are fundamentally different from homogeneous bone grafts [133].

Native cellulose, particularly BC, offers an excellent starting point due to its inherent wet-state strength [134], biocompatibility (as demonstrated in cartilaginous tissue models [135]), and nanofibrous architecture that can support osteoblast adhesion and mineralization [136]. However, its chemical inertness and lack of biological signals [137] render pure cellulose deficient in the osteoconductivity and osteoinductivity essential for bone healing [138]. Therefore, the functional enhancement of cellulose for bone repair primarily revolves around two complementary approaches: composite formulation with inorganic phases and bioactive molecular functionalization [139].

The most prevalent strategy involves incorporating calcium phosphate minerals, notably HA [140], the main inorganic component of natural bone [141]. This integration is frequently achieved through in situ biomimetic mineralization, where the abundant hydroxyl groups on nanocellulose surfaces serve as nucleation sites for HA crystal growth [142,143]. For instance, blending HA nanoparticles into cellulose matrices during processing has been shown to significantly improve the mechanical strength of the scaffold while maintaining a pore structure that meets the requirements for bone tissue regeneration [144]. This inorganic–organic synergy enhances the compressive strength and modulus of the composites, bringing them closer to the range of natural cancellous bone. Beyond HA, which primarily provides structural biomimesis, composites with bioactive glass (BG) or β-tricalcium phosphate (β-TCP) are also explored. These phases offer additional ion-releasing capabilities (e.g., Si^4+^, Sr^2+^), which can deliver biochemical cues to further stimulate osteoblast activity and angiogenesis [145,146].

Complementing mineral addition, bioactive functionalization introduces specific molecular cues to direct cellular fate [147]. The sustained release of osteoinductive growth factors, such as bone morphogenetic protein-2 (*BMP-2*), from cellulose-based matrices enhances osteogenic marker expression and mineralization in vitro [148]. Similarly, the controlled delivery of small-molecule drugs like simvastatin can upregulate osteogenic activity, albeit in a dose-dependent manner. These approaches highlight the potential of functionalized cellulose hydrogels to deliver bioactive cues for bone regeneration [149].

For complex bone defect environments, scaffolds are increasingly designed with dual functionality to simultaneously address osteogenesis and vascularization [150,151]. Several strategies have emerged to achieve this. One established approach is the co-delivery of angiogenic and osteogenic factors (VEGF and *BMP-2*) within composite matrices, which synergistically enhanced vascularized bone regeneration [152]. Beyond biochemical cues, structural design alone can confer dual bioactivity; for example, hydrogels with aligned nanopatterned surfaces and interconnected hollow channels promote cell guidance and nutrient transport, activating endogenous pathways linked to both angiogenesis and osteogenesis [153]. The most advanced designs integrate these principles into hybrid material systems. A representative example is the BC/HAP@PRP composite hydrogel, wherein BC provides a porous, stable network (structural engineering), HA enhances osteoconductivity (inorganic composite), and platelet-rich plasma ensures sustained growth factor release (molecular delivery). Together, they form a structurally robust and biologically active scaffold that promotes cell adhesion, proliferation, and osteogenic differentiation, demonstrating significant potential for bone tissue regeneration [154]. This progression—from molecular delivery and structural design to their convergence in integrated hybrid systems—epitomizes the advancing paradigm in multifunctional scaffold design for bone tissue regeneration.

**Figure 7:**
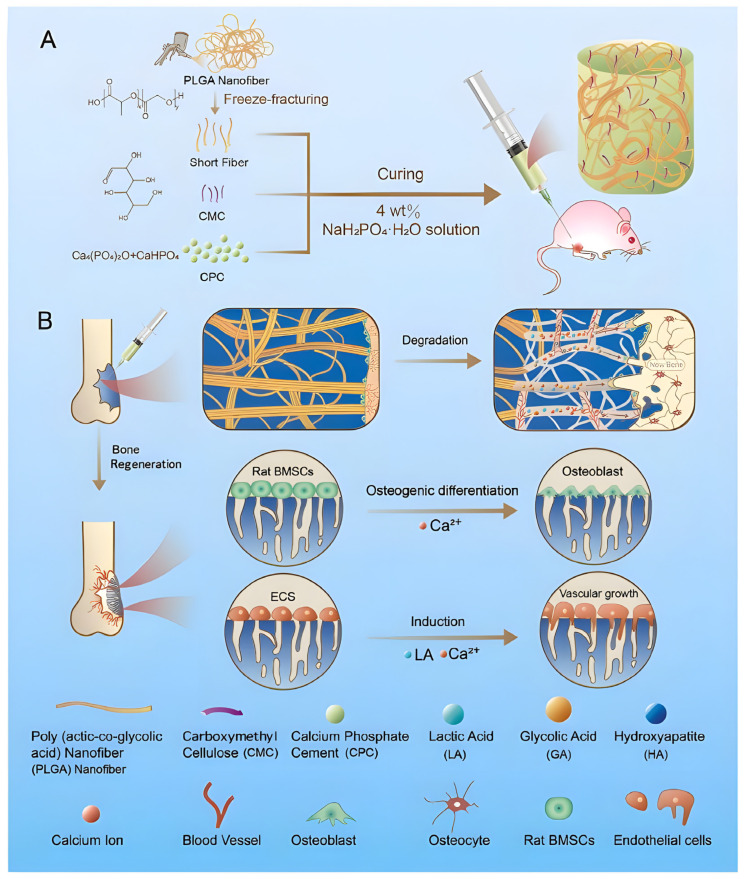
Schematic representation of fabricating C/PL/C injectable bone cement for bone regeneration. (**A**) The detailed method and process of fabricating C/PL/C. (**B**) The mechanism of bone regeneration at bone defects by the injectable bone cement [155]. Reprinted under the Creative Commons (CC) License (CC BY 4.0).

It is worth noting that this dual biological activity can also be achieved internally through a more integrated material design strategy without relying on the load of exogenous growth factors. For example, injectable PLGA nanofibers reinforced with calcium phosphate cement (C/PL/C), developed by Cai et al., can form macroporous structures that promote the growth of blood vessels through the degradation of PLGA fibers, and simultaneously release Ca^2+^ and lactic acid (Figure 7). The latter has been proven to effectively promote angiogenesis [155]. This method provides structural guidance and biochemical signals through the intrinsic design of material composition and degradation behavior, which represents an advanced paradigm with high efficiency and clinical transformation potential for vascularized bone regeneration.

Having endowed the material with the necessary bioactivity, the next critical step is to shape it into an effective three-dimensional structure. The successful translation of these engineered materials into clinically viable constructs is critically dependent on advanced fabrication technologies. Three-dimensional printing enables the construction of patient-specific scaffolds with precisely controlled, interconnected macroporosity (typically 200–500 μm), which is crucial for cell migration, tissue ingrowth, and vascularization [156,157]. Freeze-drying is another widely used method to produce highly porous sponges from cellulose composites, with porosity easily exceeding 85% [158,159]. The pore architecture can be tailored by controlling freezing parameters. For example, unidirectional freezing creates aligned microchannels that can guide cell organization [160].

Specific case studies demonstrate how distinct functionalization objectives—direct osteoinduction versus enhanced mineral affinity—guide the design of BC scaffolds, each yielding validated regenerative outcomes. The first approach focuses on imparting direct osteoinductive signals through molecular modification. Maleic acid (MA) treatment introduces carboxyl groups onto BC, yielding MA-BC that upregulates osteogenic gene expression and stimulates mineralized nodule formation in vitro. Furthermore, its successful processing into a 3D-printable bioink highlights its suitability for fabricating instructive, cell-instructive scaffolds [161]. In contrast, the second strategy emphasizes osteoconduction by optimizing biomimetic mineralization. Citrate modification enriches BC with carboxyl groups, markedly accelerating and homogenizing HA deposition from simulated body fluid. The resulting biomineralized BC (BMBC) composite exhibits enhanced compressive properties and serves as an excellent substrate for osteoblast attachment and proliferation, confirming its function as a highly osteoconductive scaffold [162]. Together, these cases illustrate that a goal-driven chemical design strategy can effectively tailor BC for specific bone regeneration mechanisms.

**Figure 8:**
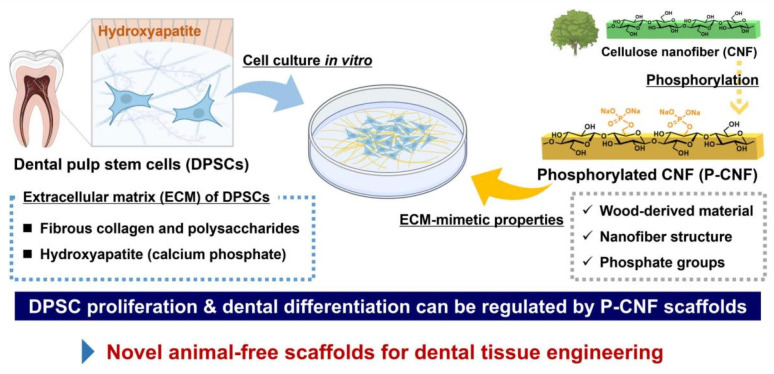
Schematic illustration of surface modification of cellulose nanofibers (CNFs) through phosphorylation, producing phosphorylated CNFs (P-CNFs) with different phosphate contents. These P-CNFs potentially mimic ECM components in tooth tissue, enabling studies of P-CNF effects on the proliferation and differentiation of human dental pulp stem cells (hDPSCs). This conceptual illustration and the graphical abstract were partially created with BioRender.com [9]. Reprinted under the Creative Commons (CC) License (CC BY 4.0).

Building on this goal-driven chemical design paradigm for bone regeneration, a parallel strategy has been successfully applied in dental tissue engineering. Phosphorylated cellulose nanofibers (P-CNFs) have been engineered to mimic the dentin ECM, thereby directing the proliferation and osteo/odontogenic differentiation of human dental pulp stem cells (hDPSCs) (Figure 8). By precisely modulating the phosphate group content, the surface chemistry and bioactivity of P-CNF scaffolds can be finely tuned, offering a versatile platform for stem cell-based pulp regeneration [9].

Addressing the more complex osteochondral unit requires sophisticated gradient or biphasic scaffold design [163]. Researchers developed an acellular bilayer scaffold based on BC, designed to recapitulate the native osteochondral tissue interface. The scaffold consists of a top layer adsorbed with chondroitin sulfate (BC-GAG) to mimic the cartilage region and promote chondrogenesis, and a bottom layer incorporated with hydroxyapatite (BC-HA) to mimic the subchondral bone and support osteogenesis. This stratified structure effectively guides stem cell differentiation toward lineage-specific phenotypes in a spatially defined manner: the BC-HA layer enhances osteogenic differentiation of human adipose-derived mesenchymal stem cells (hATMSCs), as demonstrated by increased alkaline phosphatase activity, mineralization, and calcium deposition; concurrently, the BC-GAG layer enhances chondrogenic differentiation, evidenced by elevated production of sulfated glycosaminoglycans (sGAG). By enabling spatially distinct tissue regeneration, this bilayered design promotes the simultaneous repair of both cartilage and bone layers, supporting osteochondral regeneration in vitro and in vivo [164].

To conclude, the advancement of cellulose-based scaffolds for bone and osteochondral repair highlights their efficacy through customized composite architectures, bioactive enhancements, and sophisticated fabrication techniques. These systems not only emulate the innate characteristics of native tissues, but also establish a flexible foundation for addressing intricate skeletal imperfections, thereby paving the way for applications in softer and more specialized tissue domains.

### 3.2. Soft Tissues: Skin Wound Healing and Nerve Regeneration

The regeneration of soft tissues, such as skin and peripheral nerves, presents a distinct set of challenges centered on managing a dynamic healing microenvironment and providing precise cellular guidance [138,165]. Cellulose-based scaffolds address these challenges by offering an optimal combination of biocompatibility (including favorable outcomes in peripheral nerve regeneration models [166]), tunable physical properties [167], and a nanofibrous architecture that mimics the native ECM to guide cell migration and tissue organization [168].

**Figure 9:**
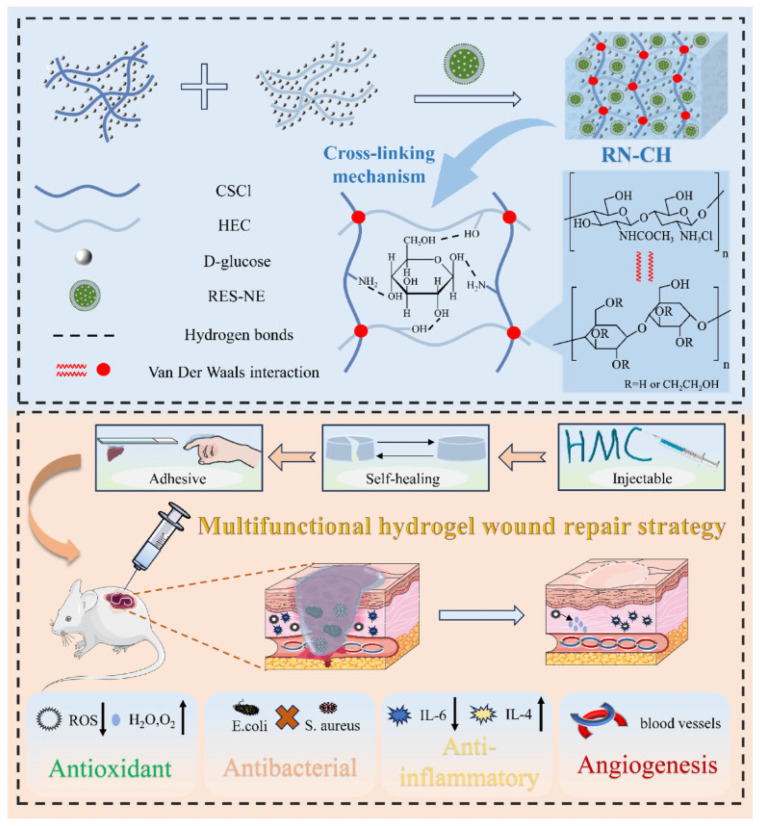
Schematic diagram showing the design principle of multifunctional hydrogel and its application in full-thickness skin wound [175]. Reprinted under the Creative Commons (CC) License (CC BY 4.0).

In skin wound healing, the primary goals are to rapidly achieve barrier restoration, manage exudate, prevent infection, and ultimately minimize scar formation while promoting the regeneration of functional dermal and epidermal structures [169]. BC possesses a unique set of intrinsic properties that align perfectly with the ideal wound dressing paradigm: its high water-holding capacity (up to 99%) maintains a moist wound environment conducive to healing [170]; its dense nanofibrillar network acts as an excellent barrier against external pathogens while allowing gas exchange [171]; and its high wet strength and flexibility enable it to conform closely to wound contours [172]. These attributes have underpinned the clinical use and commercial development of BC-based wound dressings like Biofill^®^ and XCell^®^ for burns and chronic ulcers [173]. Building upon these inherent advantages, advanced design strategies focus on integrating cellulose with other functional polymers and bioactive agents to create multifunctional dressings [174]. A prime example is a composite hydrogel (RN-CH) engineered from chitosan hydrochloride, hydroxyethyl cellulose (a cellulose derivative), and resveratrol nanoemulsion (as illustrated in Figure 9). This system demonstrates how cellulose-based platforms can be endowed with adhesive, self-healing, and injectable properties, coupled with antioxidant, antibacterial, anti-inflammatory, and angiogenic functions, collectively accelerating full-thickness wound healing in vivo [175]. This exemplifies the shift from passive coverage to active therapy.

Following this paradigm, research focuses on engineering actively functionalized cellulose dressings. A major thrust is antimicrobial functionalization to combat infection, a common complication that delays healing [176]. This is achieved by incorporating silver nanoparticles, chitosan, antibiotics, or natural extracts into the BC matrix [177]. For example, a sprayable hydrogel formed simply by mixing CNCs with the aminoglycoside antibiotic tobramycin demonstrated potent antibacterial activity and significantly accelerated the healing of infected wounds in mice [178]. To directly stimulate tissue regeneration, scaffolds are functionalized with bioactive molecules. Loading growth factors [179,180] such as VEGF or basic fibroblast growth factor (bFGF) promotes angiogenesis, while epidermal growth factor (EGF) facilitates re-epithelialization [181]. Beyond direct antimicrobial and regenerative cues, advanced designs also aim to modulate the overarching immune response [182]. For instance, nanocellulose-based hydrogels integrated with H_2_S donors such as GYY4137 have been demonstrated to promote macrophage polarization toward the pro-regenerative M2 phenotype, mitigating excessive inflammation and fostering a remodeling microenvironment [183].

Structural engineering further enhances performance. Multilayer or composite scaffolds are designed to mimic the skin’s stratified anatomy [184]. A dense BC membrane can serve as a temporary epidermis [185], while a porous BC-collagen or BC-gelatin composite layer underneath acts as a dermal template for fibroblast infiltration and vascularization [186]. Three-dimensional bioprinting enables the fabrication of such heterogeneous, cell-laden skin constructs with precise spatial control over different bioinks, potentially incorporating both fibroblasts and keratinocytes to form a bilayered living equivalent [187].

For peripheral nerve regeneration, the challenge is to bridge gaps in damaged nerves and guide the sprouting axons from the proximal to the distal stump, preventing misdirection and neuroma formation [188,189]. The key requirements for a nerve guidance conduit (NGC) include a tubular structure with appropriate porosity for nutrient exchange, topographical cues to direct axonal growth, and a supportive biochemical microenvironment [190]. Cellulose scaffolds address these needs through specific structural and functional design.

The inherent nanofibrous structure of BC closely resembles the bundled topology of the native endoneurium, providing a favorable substrate for Schwann cell adhesion and migration [191,192]. To provide physical guidance, fabrication techniques are employed to create aligned microstructures [193,194]. Exploiting analogous fibrous morphologies, anisotropic architectures can be fabricated from CNF suspensions, such as through shear-alignment methods, to create scaffolds featuring long-range, aligned microchannels. These structurally ordered substrates have been demonstrated to effectively direct neural stem cell differentiation and promote highly oriented neurite outgrowth in vitro [195]. Following a similar principle of creating aligned topographies, electrospinning can produce aligned nanofiber mats from BC/polycaprolactone (PCL) blends, which can then be rolled into tubular conduits [88].

Given that nerve tissue is electrically excitable, incorporating conductive components into cellulose-based neural guidance conduits (NGCs) is a prominent strategy to mimic the natural electrophysiological environment [196]. Composites utilizing materials such as reduced graphene oxide (rGO) or conductive polymers like polypyrrole (PPy) and poly(3,4-ethylenedioxythiophene) (PEDOT) can effectively impart electrical conductivity to the scaffolds [197,198,199]. For instance, synergistic effects in CNF/rGO/PEDOT:PSS ternary composites have been shown to achieve a high conductivity of 42.11 S/cm while maintaining mechanical strength, underscoring their potential as flexible conductive substrates for bioelectronic interfaces [200].

Bioactive functionalization complements structural guidance in neural tissue engineering. The sustained release of neurotrophic factors such as nerve growth factor (*NGF*) and glial cell line-derived neurotrophic factor (*GDNF*) from scaffold matrices provides essential chemical cues that support neuronal survival and axonal extension [201]. This concept is well-illustrated by a study in which bicomponent electrospun scaffolds were developed for the dual delivery of *GDNF* and *NGF* [202]. In this system, each factor was separately encapsulated within core-shell structured nanofibers made of PLGA or PDLLA, enabling spatially organized and temporally controlled release. By varying the ratio of the two fiber components, the release kinetics of each neurotrophic factor could be independently tuned, establishing a layered biochemical signaling environment. This orchestrated delivery not only promoted neurite outgrowth but also demonstrated a synergistic enhancement in the differentiation of PC12 cells, underscoring the potential of layered neurotrophic signaling strategies for nerve repair.

Preclinical studies in rodent sciatic nerve injury models have yielded promising outcomes [203]. Beyond material-based approaches like functionalized BC conduits—which can promote axonal regeneration and functional recovery, rivaling autograft performance in some aspects—researchers are also exploring cell-based strategies [204]. A representative example is a bio-3D conduit fabricated from human umbilical cord-derived mesenchymal stem cells (UC-MSCs). In a rat sciatic nerve model, this living conduit not only outperformed silicone controls but also achieved structural and functional outcomes comparable to autografts, including better kinematic recovery, more numerous and larger myelinated axons, and thicker myelin sheaths. Notably, it also elicited a much weaker immune response than traditional allografts, highlighting an additional immunomodulatory benefit. These collective advances underscore the broad potential of diverse bioactive conduit designs to bridge nerve defects without requiring autografts [205].

Ultimately, cellulose scaffolds serve as adaptable and customizable frameworks for soft tissue regeneration, leveraging their inherent biocompatibility, adjustable properties, and proactive biofunctionalization. The core strategies of structural direction, biochemical cues, and multifunctional engineering not only underpin success in skin and nerve repair, but also seamlessly translate to the complexities of specialized tissues, such as those discussed in the subsequent analysis of cartilage, ligament, and cardiovascular systems.

### 3.3. Specialized Tissues: Cartilage, Ligament, and Cardiovascular Applications

Beyond bone and common soft tissues, cellulose scaffolds are engineered to meet the highly specialized demands of cartilaginous, ligamentous, and cardiovascular tissues, where unique mechanical properties and biological functions are paramount.

**Figure 10:**
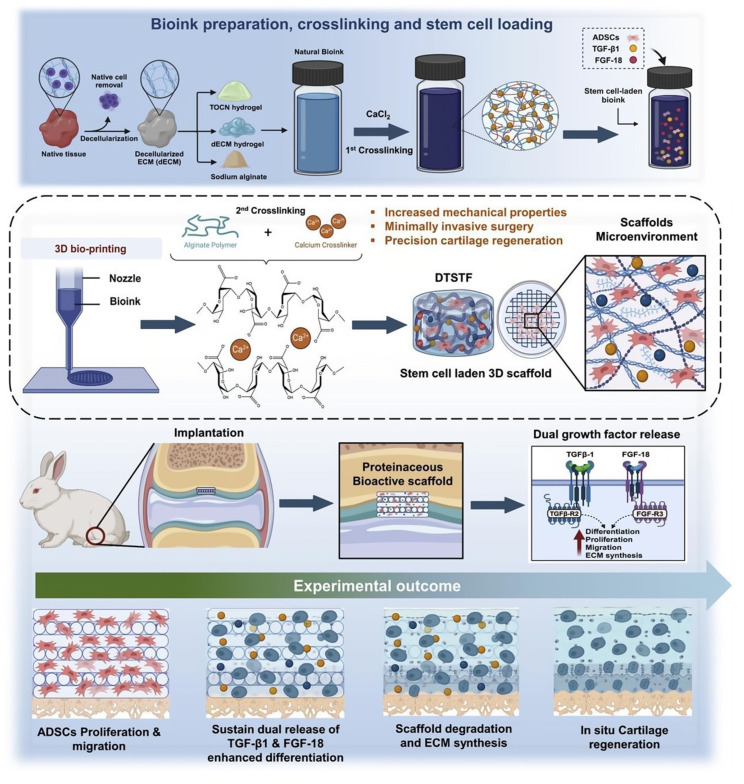
Schematic demonstration of *FGF-18*/*TGF-β1* functionalized dual-factor releasing 3D bio-printed stem cell-laden bioactive scaffolds for in situ cartilage regeneration [214]. Reprinted under the Creative Commons (CC) License (CC BY 4.0).

Cartilage tissue, particularly articular cartilage, is avascular and aneural, with limited self-repair capacity [206], placing stringent demands on a regenerative scaffold to withstand load, provide lubrication, and maintain the stable chondrocyte phenotype to prevent hypertrophy or dedifferentiation [207]. When extended to the repair of osteochondral units, these cartilage-specific demands must be integrated with osteogenic requirements in a single, seamless construct [208]. BC hydrogels are naturally suited for this application due to their high water content (simulating the hydrated cartilage matrix), excellent compressive modulus, and inherent resilience [209]. Their nanofibrous network also supports chondrocyte encapsulation and promotes the secretion of cartilage-specific ECM components like type II collagen and aggrecan [210,211]. To enhance functionality, BC is often composited with native cartilage ECM components such as hyaluronic acid (HA) or chondroitin sulfate to improve bioactivity [212]. Decellularized plant-based cellulose scaffolds, like those derived from apple hypanthium, have also shown promise by providing a unique microporous structure that favors chondrogenic differentiation of progenitor cells over fibrogenic pathways [213]. Moving beyond structural support and passive biocompatibility, advanced integrated strategies now combine precise 3D bioprinting with sophisticated growth factor delivery to create programmable microenvironments. For instance, Shanto et al. developed a proteinaceous scaffold integrating a decellularized matrix, nanocellulose (TOCN), and alginate, which was further functionalized with a dual sustained release of *TGF-β1* and *FGF-18* to actively direct adipose-derived stem cell (ADSC) fate toward effective chondrogenesis (Figure 10) [214]. This integrated approach, which combines biomimetic structure with spatiotemporally controlled biochemical signaling, exemplifies the shift toward scaffolds that actively regulate regeneration. However, primary challenges remain in achieving stable integration with native tissue in osteochondral defects and ensuring long-term mechanical durability under physiological loading [215].

**Figure 11:**
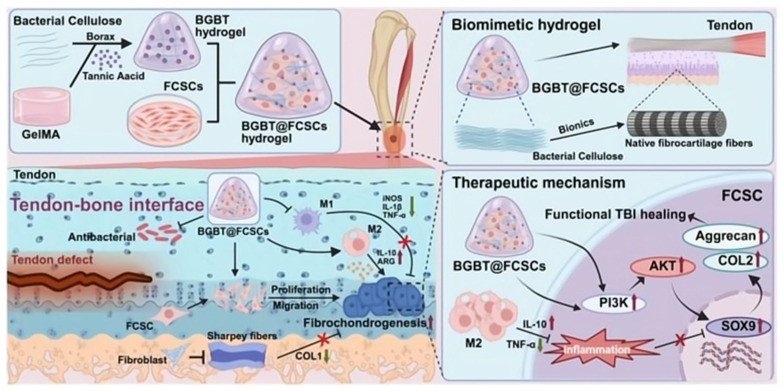
Fabrication and application of BGBT dual-crosslinked network hydrogel loaded with FCSCs for treatment of TBI in rats. Created with BioRender.com. FCSCs, fibrocartilage stem cells; TBI, tendon-to-bone interface [221]. Reprinted under the Creative Commons (CC) License (CC BY 4.0).

Ligament and tendon regeneration demands scaffolds with high tensile strength, toughness, and anisotropy to effectively transmit uniaxial mechanical forces [158,216]. Cellulose micro- and nanofibers are well-suited for this purpose due to their intrinsic high tensile strength. Electrospinning is a key technique for producing aligned nanofiber mats from cellulose derivatives or composites, which mimic the aligned collagen fibril structure of native tendons and ligaments. For instance, incorporating CNCs into aligned PCL/chitosan nanofibers increased toughness by 85% and achieved tendon-relevant mechanical properties (σ = 39.3 MPa, E = 540.5 MPa), while promoting tenocyte alignment [217]. Similarly, nanofibrous patches combining nanocellulose (NFC) and lysozyme nanofibers (LNFs) exhibited high tensile strength (Young’s modulus ≥ 3.7 GPa), along with antioxidant and antimicrobial activities, and promoted fibroblast migration. These vacuum-filtered NFC/LNFs constructs demonstrate the potential of bio-based nanofibrous designs for load-bearing and bioactive tissue regeneration [218]. Such oriented fibers provide contact guidance for tenocytes and fibroblasts, encouraging cell alignment and organized ECM deposition. To achieve greater mechanical integrity, continuous cellulose-based fibers can be produced by wet-spinning and then woven or braided into bundles, better resembling the hierarchical architecture of ligaments [158,216]. One study showed that applying an AC electric field during wet-spinning significantly enhanced CNF alignment, leading to improved Young’s modulus (up to 28 GPa) and toughness (15 MJ/m^3^) [219]. These strong, continuous nanocellulose fibers are ideal for weaving into hierarchical ligament-like bundles. Advanced strategies to further stimulate functional regeneration include functionalizing scaffolds with tenogenic growth factors or using composite bioinks for 3D bioprinting. For example, sequential delivery of *FGF-2* followed by *TGF-β3* was shown to significantly enhance teno/ligamentogenic gene expression in periodontal ligament stem cells (PDLSCs), while suppressing the osteogenic marker *RUNX2* [220]. This supports the use of sequential growth factor delivery to promote targeted differentiation and minimize complications such as heterotopic ossification in tendon and ligament regeneration. Beyond growth factor delivery, recent strategies integrate structural, functional, and biological cues within a single system. As illustrated in Figure 11, a multidimensional biomimetic approach employing a dual-crosslinked BGBT hydrogel loaded with fibrocartilage stem cells (FCSCs) has been developed for tendon-to-bone interface regeneration. This system exemplifies how cellulose-based BC networks can be engineered to mimic native tissue architecture while delivering cells and modulating the regenerative microenvironment [221].

In cardiovascular tissue engineering, a key challenge lies in fabricating small-diameter vascular grafts that resist thrombosis and intimal hyperplasia while matching the mechanical compliance of native arteries [222,223]. BC offers a distinctive approach through its unique biosynthetic molding capability. Using the BASYC^®^ (Bacterial Synthesized Cellulose) technique, sterile BC tubes can be directly cultured in silicone mold bioreactors, producing seamless grafts with a native-like nanofibrous wall structure [224]. These tubes demonstrate favorable burst pressure and suture retention strength [225], while their smooth inner surface exhibits low thrombogenicity. The porous wall further allows potential endothelial cell seeding or transmural tissue integration [226]. For myocardial repair following infarction, cellulose-based implantable patches are designed to mechanically support the weakened heart wall, deliver therapeutic agents, and potentially carry cells. Patches made from oxidized nanofibrillated cellulose (OxNFC) and lysozyme amyloid nanofibrils (LNFs) have shown suitable mechanical properties, antioxidant activity, and the capacity to act as carriers for RNA-loaded nanoparticles. The patch serves as a platform for the localized delivery of siRNA, enabling targeted gene silencing to modulate the post-infarct microenvironment for potential therapeutic benefit [61]. Major hurdles in cardiovascular applications include ensuring long-term patency and anti-thrombogenicity of vascular grafts, achieving electrical integration of cardiac patches with host tissue, and fine-tuning the degradation profiles of these supports to enable gradual load transfer to regenerating tissue [227].

Collectively, the utilization of cellulose scaffolds in tissue regeneration embodies a purposeful ‘design-to-function’ approach, as summarized in Table 3, achieved through deliberate material selection, precise chemical modifications, composite strategies, and advanced manufacturing for structural precision. This evolution transmutes cellulose from a basic biopolymer into an array of specialized regenerative tools, as evidenced by advancements in bone, skin, neural, cartilage, and cardiovascular contexts. Nonetheless, the transition toward clinical adoption necessitates a critical evaluation of ongoing translational challenges, which will be explored in the following discourse on hurdles and future directions.

## 4. Perspective

Through molecular design, composite engineering, and advanced fabrication, cellulose-based scaffolds have demonstrated considerable and versatile potential in preclinical models for tissue repair, ranging from bone regeneration to wound healing [126,228,229]. Notably, for some applications, this foundation has begun to drive clinical translation, with BC formulations for wound management emerging as a frontrunner. Clinical trials on BC films and composites have confirmed their safety and efficacy in humans. For instance, BC films facilitated the management of post-surgical incisions in varicose vein surgery, achieving comparable healing with significantly reduced removal pain versus conventional dressings [230]. Similarly, in randomized trials on complex ischemic wounds following lower-limb revascularization, BC-based dressings demonstrated both feasibility and improved healing outcomes [231].

However, it is critical to distinguish these successes in relatively straightforward applications from the broader, more complex landscape of regenerative medicine. The leap from targeted wound care or such targeted applications [232] to the regeneration of structurally and functionally complex tissues presents fundamentally different and more severe translational challenges. These pervasive barriers currently constrain the clinical adoption of cellulose scaffolds across most of their proposed uses. This section therefore examines these persistent translational barriers and explores emerging trends that leverage cellulose’s unique properties to address them.

### 4.1. Performance Optimization: Matching Material to Biology

The clinical translation of cellulose scaffolds requires precise alignment of their inherent properties with dynamic biological processes [232]. A primary challenge in utilizing BC for TE is engineering controllable degradation kinetics. The inherent structural stability of native BC leads to extremely slow and unpredictable in vivo degradation, which fundamentally contradicts its role as a temporary scaffold designed to resorb in sync with new tissue formation. To address this, deliberate material engineering is essential [233,234,235,236]. For example, a study demonstrated that under simulated physiological conditions (PBS with lysozyme), a BC scaffold dependent on physically adsorbed cellulose achieved only 25.84% cellulose hydrolysis over 30 days, quantitatively underscoring its slow degradation profile. In contrast, an engineered BC composite featuring a controlled-release mechanism with covalently immobilized cellulose within a gelatin matrix exhibited a substantially higher hydrolysis yield of 65.86% within the same period. This system not only accelerated degradation, but also enabled a tunable degradation profile, as the final hydrolysis yield directly correlated with the enzyme loading (ranging from 7.08% to 65.86%). This work demonstrates a targeted engineering strategy to overcome the degradation-rate limitations of conventional BC-based scaffolds [237]. Mismatched degradation can result in premature failure, impaired remodeling, or chronic inflammation [238,239,240]. Strategies like periodate oxidation [241,242] or polymer blending [243,244] aim to accelerate degradation, but often lack predictable profiles under physiological conditions and may compromise initial mechanics [245], necessitating more sophisticated degradation engineering.

Concurrently, ensuring long-term immuno compatibility is equally critical. Chemical modifications and composite integration introduce variables that can trigger adverse immune responses [246,247,248]. The scaffold’s surface properties (nanotopography, charge, wettability) continuously dialogue with the host immune system, influencing macrophage polarization toward pro-regenerative (M2) versus pro-inflammatory (M1) phenotypes [249]. However, systematic mapping of how specific cellulose chemistries and topographies dictate this response remains nascent [250]. For instance, certain surface modifications aimed at enhancing adhesion have inadvertently intensified early inflammation, highlighting the delicate balance required [251,252]. Comprehensive long-term in vivo immunophenotyping studies are still needed to predict implant acceptance.

### 4.2. Product Manufacturing: From Scalability to Personalization

Transitioning from laboratory prototypes to reliable, standardized products faces significant manufacturing challenges, with reproducibility and scalability representing a primary bottleneck. Advanced techniques such as 3D bioprinting and electrospinning are highly sensitive to processing parameters [253,254], leading to batch-to-batch inconsistencies in critical features such as pore architecture, fiber diameter, and mechanical properties [255]. Scaling these intricate processes to robust, Good Manufacturing Practice (GMP)-compliant production is a significant challenge [256], compounded by the inherent variability in biological raw materials (e.g., plant feedstocks, bacterial fermentation yields), which complicates the standardization of raw nanocellulose and affects final product consistency.

Looking beyond standardization, the future of cellulose scaffolds lies in personalization and digital integration. This extends past anatomical matching via 3D printing, to include tailoring the cellulose material itself; for instance, selecting specific sources (tunicate- vs. plant-derived CNC) or degrees of oxidation to interact with a patient’s unique immune profile or metabolic state [257]. Multi-material cellulose bioprinting can further enable the seamless integration of regions with distinct mechanical or biochemical properties within a single implant [258]. Moreover, scaffolds are evolving from passive structures into active monitoring devices. Incorporating conductive cellulose composites (e.g., with graphene or PEDOT: PSS) could allow real-time monitoring of local electrical activity or mechanical strain, providing valuable feedback on the healing process [5,22]. Underpinning these advances is the growing role of digital tools. Computational modeling and machine learning are crucial for predicting the complex interplay between design parameters, manufacturing processes, and in vivo outcomes, enabling in silico optimization [26,259]. The establishment of open-source, standardized databases for cellulose scaffold properties and biological performance data is an urgent community need to accelerate this data-driven progress.

### 4.3. Intelligent Responsive Design and Translation

To dynamically interact with the regenerative microenvironment, next-generation scaffolds must exhibit intelligent, stimuli-responsive capabilities [260]. Advanced spatial functionalization, which includes strategies such as the in situ incorporation of bioactive peptides during BC fermentation [261] and the directional assembly of plant-derived nanocellulose [13,20], enables the precise integration of biochemical cues into the scaffold architecture. This level of functional sophistication, however, intensifies the complexity of biocompatibility assessment within the regulatory framework for combination products [262].

A key trajectory is the development of stimuli-responsive cellulose systems that undergo programmable changes in response to physiological cues such as specific enzymes, pH shifts, or reactive oxygen species (ROS) [117]. This intelligence allows for on-demand drug release or spatially/temporally targeted degradation. Four-dimensional bioprinting with cellulose-based inks embodies this direction, where printed constructs can morph into predefined, complex shapes after implantation, facilitating minimally invasive delivery and optimal anatomical adaptation [263].

The development of such advanced scaffolds underscores the overarching significance of the regulatory pathway, which itself constitutes a major translational hurdle, especially for products classified as combination products or Advanced Therapy Medicinal Products (ATMPs) [208]. Incorporating cells, genetic material, or complex bioactives invites stringent scrutiny [262]. Robust preclinical evidence from clinically relevant models is essential [264], yet many studies remain confined to small rodents and short-term endpoints, which are insufficient for predicting long-term performance in load-bearing human tissues [265]. Long-term human studies, such as one with a mean 4.5-year follow-up identifying key predictors of osteochondral graft survival [266], are vital for bridging this gap. The lack of globally harmonized, application-specific performance standards further hampers comparative analysis and consistent regulatory evaluation [259].

The journey of cellulose from a structural biopolymer to a potential cornerstone of regenerative medicine encapsulates the promise of bioinspired innovation. By embracing a strategic, collaborative, and translation-focused approach centered on elucidating bio-instructive mechanisms, pioneering intelligent cellulose chemistry, building translational infrastructure, and fostering convergent collaboration, the field can transform this vast potential into a new generation of intelligent, effective, and accessible clinical solutions for tissue regeneration.

## 5. Conclusions

This review has systematically charted the transformative journey of cellulose from a foundational biopolymer to a programmable platform for advanced tissue scaffolds. By integrating rational material design—spanning chemical modification, composite formulation, and bioactive functionalization—with precision manufacturing techniques, cellulose-based scaffolds can now be engineered to meet the specific structural and biological demands of diverse tissues, from bone and skin to nerve and cardiovascular systems. The central imperative now is a paradigm shift from a material-centric view to a host-instructive paradigm, where scaffolds are designed as dynamic systems that actively orchestrate endogenous healing processes—such as immunomodulation, vascularization, and innervation—rather than serving as static structural supports. Achieving this vision requires moving beyond isolated advances toward a convergent, translation-focused framework. By bridging the fundamental understanding of cellulose bio-interactions with intelligent design and clinical insight, the field can unlock the full potential of this versatile biomaterial. Ultimately, this progression promises to translate the engineered promise of cellulose into a new generation of intelligent, effective, and clinically viable solutions for regenerative medicine.

## 6. Methodology for Literature Search and Selection

To ensure a comprehensive and balanced overview of the field, a structured approach was employed to identify and select the relevant literature for this review.

Search Strategy: Electronic searches were conducted in the core databases of PubMed, Web of Science, and Scopus.

Time Frame: The search focused on the recent and rapidly evolving period from January 2015 to December 2025, to capture the latest advances in cellulose scaffold engineering.

Keywords: A combination of the following key terms and their variants was used: (“cellulose” OR “nanocellulose” OR “bacterial cellulose”) AND (“scaffold” OR “hydrogel” OR “3D print”) AND (“tissue engineering” OR “bone regeneration” OR “wound healing” OR “nerve regeneration” OR “cartilage” OR “vascular”).

Inclusion Criteria: Studies were included if they: (1) primarily focused on the design, fabrication, or functionalization of cellulose-based scaffolds; (2) reported experimental outcomes relevant to tissue engineering and regenerative medicine; (3) were published in peer-reviewed English-language journals.

Selection Process: The initial search yielded approximately 700 records. After removing duplicates, titles and abstracts were screened for relevance. The full texts of potentially eligible articles were then assessed against the inclusion criteria. Additionally, the reference lists of key articles and relevant reviews were manually searched to identify further significant studies.

## Figures and Tables

**Table 1:** Summary of representative cellulose-based scaffolds: composition, properties, and tissue regeneration.

Cellulose Source/Type	Functionalization Strategy	Fabrication Technique	Key Structural/ Properties	Tissue Regeneration	Ref.
TEMPO-CNCs/Carbon Dots	Fluorescent functionalization (for tracking)	DLP 3D printing	Photocurable resin, precise microstructures, fluorescence	Skin and vascular regeneration	[71]
Carboxylated CNCs	Nano-reinforcement and crosslinking site	Extrusion 3D printing	Enhanced shear-thinning, mechanical strength, shape fidelity	General tissue engineering	[43]
Bacterial Cellulose (BC)	Oxidation, cationic PU micelle and CD grafting	In situ biosynthesis + chemical crosslinking	Dual-network, sustained drug release, antibacterial, cytokine recruitment	Urethral regeneration	[70]
Cellulose Nanofibers (CNFs)	Phosphorylation	Casting	Reduced fiber size/crystallinity, tuned wettability for protein/cell adhesion	Dental pulp stem cell culture	[9]
Gelatin/Nanocellulose/nHA/Simvastatin	Composite formulation and drug loading	Freeze-drying + chemical crosslinking (glutaraldehyde)	Highly porous sponge, osteoconductive and osteoinductive	Bone regeneration	[17]
TOBC (m-TOBC)/GelMA/DMOG-loaded MSNs	Enzyme mineralization and bioactive loading	Extrusion 3D printing	Biomimicked bone ECM, improved rheology/mechanics, angiogenic drug release	Bone regeneration	[38]
CNF/Mg-Fe LDH/RA and SHH	Composite formulation and growth factor loading	Anisotropic freeze-drying	Aligned microchannels, sustained factor release	Neural regeneration	[20]
TOCNF/rGO (PEI-modified)	Composite formulation and surface charge modification	Extrusion 3D printing	Conductive, good printability and mechanical recovery, supports neural cell growth	Neural regeneration	[11]
BC/PCL	Polymer blending	Electrospinning	Nanofibrous mesh with hollow microbeads, enhanced cell adhesion/proliferation	Neural regeneration	[88]
BC/Poly(3,4-ethylenedioxythiophene)-SNFs	Sulfonation and in situ polymerization	In situ assembly + rolling/coating	Conductive composite membrane, layered structure, suitable for nerve conduits	Peripheral nerve repair	[22]
CMC/CMCS/Gelatin/Algal Extracts	Composite formulation and bioactive incorporation	Freeze-drying + chemical crosslinking (EDAC)	Porous hydrogel sponge, enhanced wound healing properties	Skin wound healing	[55]
Cellulose Acetate (CA)	N-halamine compound grafting (MDI)	Electrospinning	Antimicrobial nanofibrous mats, durable, non-leaching	Antimicrobial wound dressing	[44]
PLA/CNF (from *Pennisetum purpureum*)	Composite formulation	Solvent casting and salt leaching	Highly porous (>76%), improved compressive strength and hydrophilicity	Bone regeneration	[12]
PHB/Chitosan/CNC	Composite formulation	Electrospinning	Enhanced tensile strength/modulus; Osteogenic marker upregulation	Bone regeneration	[58]
BC/BAM (Bladder Acellular Matrix)	Composite formulation	Freeze-drying + chemical crosslinking (EDC/NHS)	Biomimetic composition (collagen, GAGs, VEGF), enhanced mechanical strength	Urethral regeneration/Angiogenesis	[95]
Oxidized BC (OBC)/Soy Protein Isolate (SPI)	Selective oxidation and protein composite	Laser perforation + oxidation + compositing	Improved biocompatibility, cell guidance, degradability	Urethral regeneration	[116]

**Table 2:** Comparative summary of key performance metrics for representative cellulose-based scaffolds.

Scaffold Composition	Target Tissue	Mechanical Property	Porosity (%)	Pore Size (μm)	Degradation Time	Performance Highlight	Ref.
Gelatin, Bacterial Nanocellulose, Nanohydroxyapatite, Simvastatin	Bone tissue	NR	NR	BNC-G group: 211–244 μm average; No pores > 300 μm	~21% mass loss in 4 weeks for BNC-G-nHA-Sim scaffold (in vitro, PBS)	Excellent biocompatibility; Significantly enhanced osteogenic differentiation of BMSCs; Sustained simvastatin release over 216 h.	[17]
GelMA/m-TOBC/DMSN hydrogel	Bone tissue	Compressive modulus: ~15.6 Kpa; Enhanced storage modulus (G’)	NR	NR	~20% mass loss after 7 days (in vitro, collagenase II)	Biomimetic bone ECM; Improved printability and mechanics; Synergistic release of osteogenic ions (Ca^2+^, PO_4_^3−^) and angiogenic drug (DMOG).	[38]
PHB-chitosan/CNC (3 wt% CNC)	Bone tissue	Tensile strength: 4.52 MPa; Modulus: 130.29 MPa	NR	NR	~35% weight loss after 100 days (PBS)	Significant osteogenic gene upregulation (*OPN*: 10.7-fold, *ALP*: 4.1-fold); High cell viability (91.5%); Excellent bioactivity (Ca/P ratio ~ 1.73); Enhanced hydrophilicity and surface roughness.	[58]
GelMA, PEGDA, T-CNC@CDs, LAP	Skin, Blood vessel, Muscle	Elastic modulus: ~13 ± 4.2 Kpa (for GPCD hydrogel)	NR	GPCD: 166 ± 20 μm; GM: 82 ± 13 μm; GPD: 40–120 ± 10 μm.	Short-term: ~35–48% weight loss in 5 h (PBS/trypsin)	>95% cell viability; Upregulated gene expression (*COL1A*: 6.74-fold, *KRT1*: 4.27-fold); 30-day cell tracking; Stable fluorescence (pH 6.5–9.5); High-resolution DLP printability.	[71]
CMC/CMCS/Gelatin hydrogel loaded with 1% *Arthrospira platensis* (AP) and/or 1% *Chlorella vulgaris* (CV) extracts.	Skin (Wound healing)	NR	NR	~37–105 μm (AP reduced pore size more than CV).	~24–48 h (in PBS; AP/CV groups dissolved within 24 h, base hydrogel degraded 32% in 48 h).	Synergistic effects; Highest wound closure rate (92%) at 14 days in a rat model.	[55]
CNF/Chitosan aerogels (CNF1/2 with Ch)	Skin (Wound healing)	NR	pore volume 0.122–0.357 cm^3^/g	~0.003 μm (3.0–3.5 nm)	NR	High biocompatibility and strong antibacterial activity against *S. aureus* and *E. coli*.	[125]
Bilayer: RC/Quaternized CS base layer + Collagen/HA top layer.	Skin (Wound healing)	Ultimate tensile strength (UTS): 2.29 MPa (for crosslinked bilayer scaffold).	>80%	NR	Degradation observed over 10 days in mild acidic pH (4.5–5.6)	Antibacterial (vs. *E. coli*); Enhanced angiogenesis (9.76-fold increase in *VEGF-A* gene expression); Promoted cell migration (100% scratch closure); Stimulated collagen secretion.	[126]
TOCNF/rGO (PEI-modified)	Neural tissue	379.94–1267.28 Kpa	NR	NR	14 days (mass loss decreased with rGO content)	>90% printing fidelity; Conductive (up to 0.21 S/m); Supports astrocyte viability and alignment.	[11]
CNF/Mg–Fe LDH loaded with RA/SHH	Neural tissue	Similar modulus to skin tissue (for CNF film); Aligned microchannel structure provides topological guidance	NR	Homogeneously distributed pores; Aligned microchannels present	NR	Promotes aligned axonal growth; Enhances neuron/oligodendrocyte differentiation; Suppresses astrocytes; Restores motor function and electrophysiology via *RhoA*/*Rock*/*Myosin II* pathway.	[20]
BC/PCL (50:50 wt.%) blend, electrospun	Neural tissue	Max. tensile strength ~30 MPa (vs. ~14.6 MPa for pure PCL)	NR	Fiber diameter: 70–120 nm; Hollow beads: 100 nm–1.6 μm	NR	Enhanced fibroblast adhesion/proliferation vs. pure PCL; Supported DRG neurite outgrowth and alignment.	[88]
BC/PEDOT-SNFs (BPS) composite membrane	Peripheral nerve	Tensile strength: 5.80 ± 0.14 MPa (BPS 10–10); Young’s modulus: ~0.33 MPa	~97%	NR	NR	High conductivity (up to 10^−2^ S/cm); Excellent biocompatibility; Promotes ADSCs adhesion; Improves peripheral nerve regeneration in vivo.	[22]
BC/BAM composite	Urethral tissue	Compressive stress: 33 → 74 Kpa; Young’s modulus: 12 → 90 Kpa	>85%	187 ± 59 μm (BC_0.5_/BAM_0.5_)	NR	Promoted angiogenesis and epithelialization; Achieved stricture-free repair in rabbit model.	[95]
DMBC/SPI (Double-Modified BC with Soy Protein Isolate)	Urethral tissue	~1.58 ± 0.12 MPa (DMBC/SPI1)	NR	200–300 μm (laser-drilled macropores)	~60 days (50.03% mass loss in PBS)	Promotes aligned cell growth; Supports smooth urethral regeneration with mild inflammation.	[116]
MCC scaffold embedding PLGA/KGN microspheres (MCC/PKGN)	Cartilage	4.53 MPa	NR	200–500 μm	Partial degradation (7.73% at 8 weeks in vitro)	Sustained KGN release promotes BMSC migration; Chondrogenic differentiation; Enhanced cartilage regeneration in vivo.	[123]
EDC/NHS-crosslinked macroporous bacterial cellulose (cMP-BC)	Cartilage	Enhanced compressive strength and shape recovery in wet state; Stable 3D network in aqueous environment	NR	64–195 μm (tunable via BC concentration)	NR	Excellent biocompatibility; Promoted chondrocyte infiltration and cartilage-specific ECM deposition; Mechanically robust and shape-recoverable.	[127]

(NR = Not Reported).

**Table 3:** Application-specific requirements and corresponding scaffold design solutions.

Target Tissue	Core Functional Requirements	Representative Cellulose Scaffold Design	Key Design Rationale and Features	Ref.
Bone	Mechanical support and load-bearing; Osteoconductivity and Osteoinductivity; Vascularization support	Composite Scaffold (Gelatin/Nanocellulose/nHA/Simvastatin)	Composite formulation and drug loading: nHA provides osteoconduction; Nanocellulose reinforces; Simvastatin release induces osteogenesis.	[17]
		Mineralized TOBC/GelMA/DMOG-loaded MSNs	Bioactive mineralization and angiogenic loading: TOBC reinforces and mimics bone ECM; In situ mineralization enhances mechanics; DMOG delivery promotes vascularization.	[38]
Skin Wound	Moisture management and exudate absorption; Barrier against infection; Promotion of re-epithelialization and angiogenesis	Bilayer RC/Quaternized CS-HA/Collagen Scaffold	Bilayer structure and multifunctional composite: Dense RC/CS layer provides antibacterial barrier; Porous Collagen/HA layer promotes cell migration.	[126]
		CMC/CMCS/Gelatin/Algal Extracts	Composite hydrogel and bioactive incorporation: Polysaccharide matrix ensures high absorbency; Natural extracts enhance healing.	[55]
		TEMPO-CNF-based Bioaerogels (with Chitosan, Gelatin, Alginate)	Tunable absorption and antibacterial activity: High porosity and carboxyl groups enable exceptional fluid uptake (WAV); CNF/Chitosan composites show strong antibacterial effects.	[125]
Neural Tissue	Topographical guidance for axonal growth; Support for Schwann cell activity; Neurotrophic factor delivery	CNF/Mg-Fe LDH/RA and SHH Scaffold	Anisotropic structure and growth factor loading: Aligned microchannels direct neurite extension; LDH enables sustained factor release;	[20]
		BC/PCL (50:50 wt.%) nanofibrous scaffold with embedded hollow micro/nanobeads	Anisotropic fibers guide neurite growth. Hollow beads are drug-loadable; BC provides hydrophilicity and biocompatibility; PCL enhances mechanical strength; Blend enhances biocompatibility and neural growth.	[88]
Cartilage	Compressive strength and elasticity; Support for chondrogenic phenotype; Lubrication and integration	MCC Scaffold embedding PLGA/KGN Microspheres	Controlled drug delivery: MCC provides support; PLGA microspheres enable sustained release of chondroinductive agent (KGN).	[123]
		Macroporous BC Scaffold (MP-BC)	Macroporosity and mechanical robustness: Crosslinking enhances compressive strength and shape recovery; Interconnected macropores facilitate cell infiltration and ECM deposition.	[127]
Cardiovascular (Vascular Graft)	Suture retention and burst pressure; Anti-thrombogenicity; Compliance matching native vessel	BC/Bladder Acellular Matrix (BAM)	Biomimetic composite: BC provides strength; BAM provides natural ECM to enhance endothelialization.	[95]
		In situ Biosynthesized BC Tubular Graft (BASYC^®^)	Seamless nanofibrous structure: Native BC network provides high wet strength and a smooth, low-thrombogenicity lumen.	[224]

## Data Availability

The original data used in this study are all publicly available data from the Web of Science Core Collection.
